# Littré’s hernia within a recurrent inguinal hernia sac: an unusual cause of acute abdomen in the elderly—a case report

**DOI:** 10.1093/jscr/rjag760

**Published:** 2026-08-31

**Authors:** Francisco Gil Quintero, Cristina Judith Padilla Herrera, Andrés Felipe Ramírez Carrillo, Julián Camilo Ariza Rocha

**Affiliations:** Department of General Surgery, Clínica Santa María del Lago, Calle 73A #76-66, Engativá District, Bogotá, D.C. 111051, Colombia; Department of General Surgery, Clínica Santa María del Lago, Calle 73A #76-66, Engativá District, Bogotá, D.C. 111051, Colombia; Department of General Surgery, Clínica Santa María del Lago, Calle 73A #76-66, Engativá District, Bogotá, D.C. 111051, Colombia; General Surgery Rotation, Clínica Santa María del Lago, Calle 73A #76-66, Engativá District, Bogotá, D.C. 111051, Colombia

**Keywords:** inguinal hernia, Meckel’s diverticulum, intestinal obstruction, hernia repair, elderly, acute abdomen

## Abstract

Littré’s hernia is defined as the protrusion of a Meckel’s diverticulum through a hernia defect; it represents fewer than 0.1% of complicated hernias, and its presentation as a recurrent hernia in the elderly is exceptional. We report an 81-year-old man with a previous open right inguinal herniorrhaphy who presented with abdominal pain, vomiting, and a non-reducible right inguinal mass consistent with intestinal obstruction. Exploratory laparotomy revealed a strangulated recurrent right inguinal hernia Nyhus IVB/European Hernia Society (EHS) L3R (lateral inguinal hernia, size 3 [>3 cm], recurrent), containing a 10-cm Meckel’s diverticulum located 80 cm from the ileocaecal valve, together with adjacent ileal involvement (mixed Littré’s hernia). Segmental small-bowel resection with side-to-side stapled anastomosis and primary tissue repair without mesh were performed. The patient was discharged on postoperative Day 4 and recovered uneventfully. Early intraoperative recognition and individualised surgical management are essential when contamination contraindicates prosthetic reinforcement.

## Introduction

Littré’s hernia is the presence of a Meckel’s diverticulum within a hernia sac, with or without other viscera. First described by Alexis Littré in 1700, fewer than 100 cases have been reported worldwide [1–3]. Meckel’s diverticulum is the most common congenital anomaly of the gastrointestinal tract, with an estimated prevalence of 2%; only 4%–9% of carriers become symptomatic during their lifetime [4].

Littré’s hernia accounts for ~0.09% of incarcerated or strangulated hernias and affects fewer than 1% of patients with Meckel’s diverticulum [2, 5]. In the systematic review by Schizas *et al*., the femoral location was most frequent (39.6%), followed by inguinal (34%) and umbilical (11.3%), with marked right-sided predominance (up to 98% of inguinal cases) [6]. The largest registry, by Răcăreanu *et al*., identified 98 cases with a mean age of 56.7 years, intestinal obstruction in 35.6% and overall mortality of 11.2% [7].

Preoperative diagnosis is uncommon (5%–10% of cases) because the presentation is indistinguishable from that of any complicated hernia [8, 9]. Strangulation within a recurrent sac in an elderly patient poses additional challenges, particularly when choosing a repair in a contaminated field. We report such a case and analyse its clinical, diagnostic, and surgical implications.

## Case report

An 81-year-old man with arterial hypertension, hypothyroidism, and a previous open right inguinal herniorrhaphy 4 years earlier presented with a 1-day history of severe generalised abdominal pain (predominantly right iliac fossa and flank), a non-reducible right inguinal mass, food-content vomiting, 48 h of obstipation, asthenia, and adynamia, without fever.

**Figure 1:**
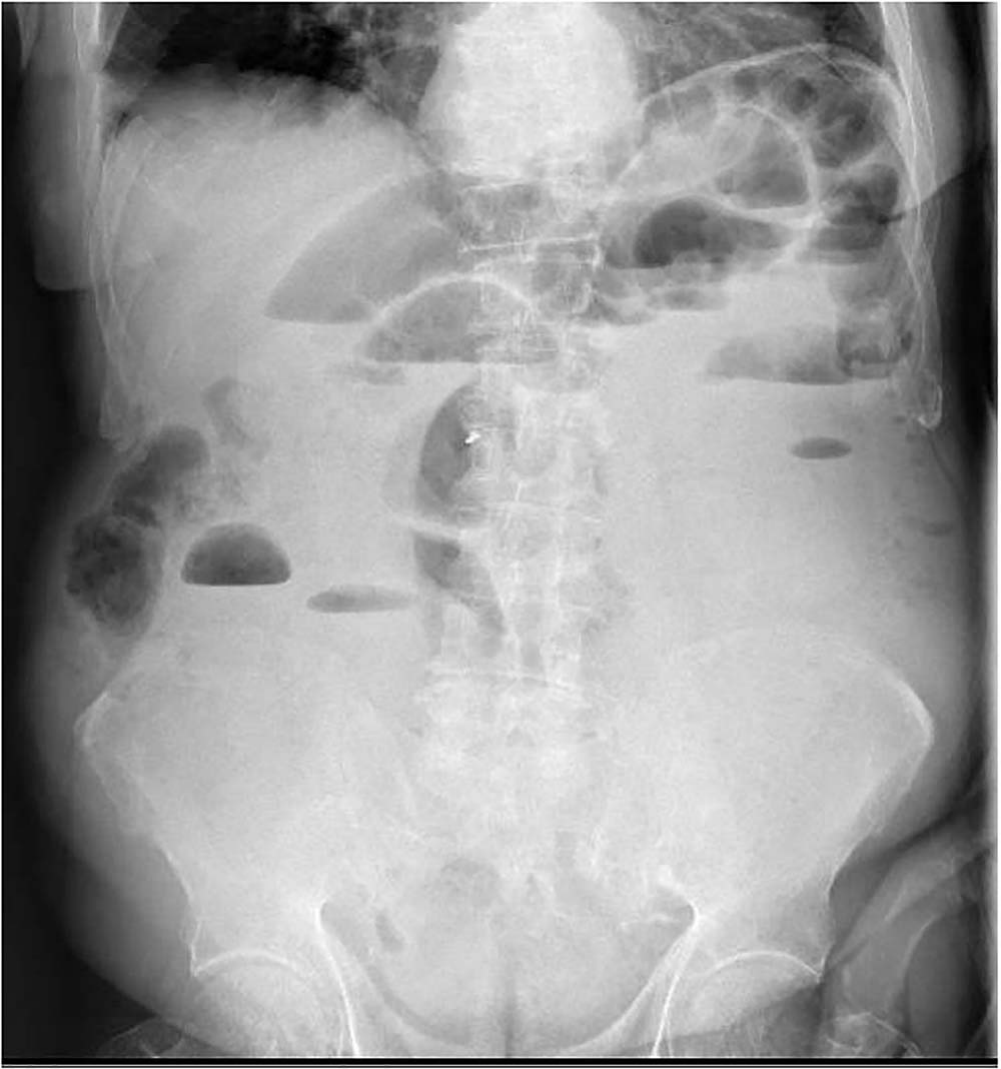
Upright plain abdominal radiograph showing distended small-bowel loops with multiple stepped air–fluid levels, consistent with intestinal obstruction.

**Figure 2:**
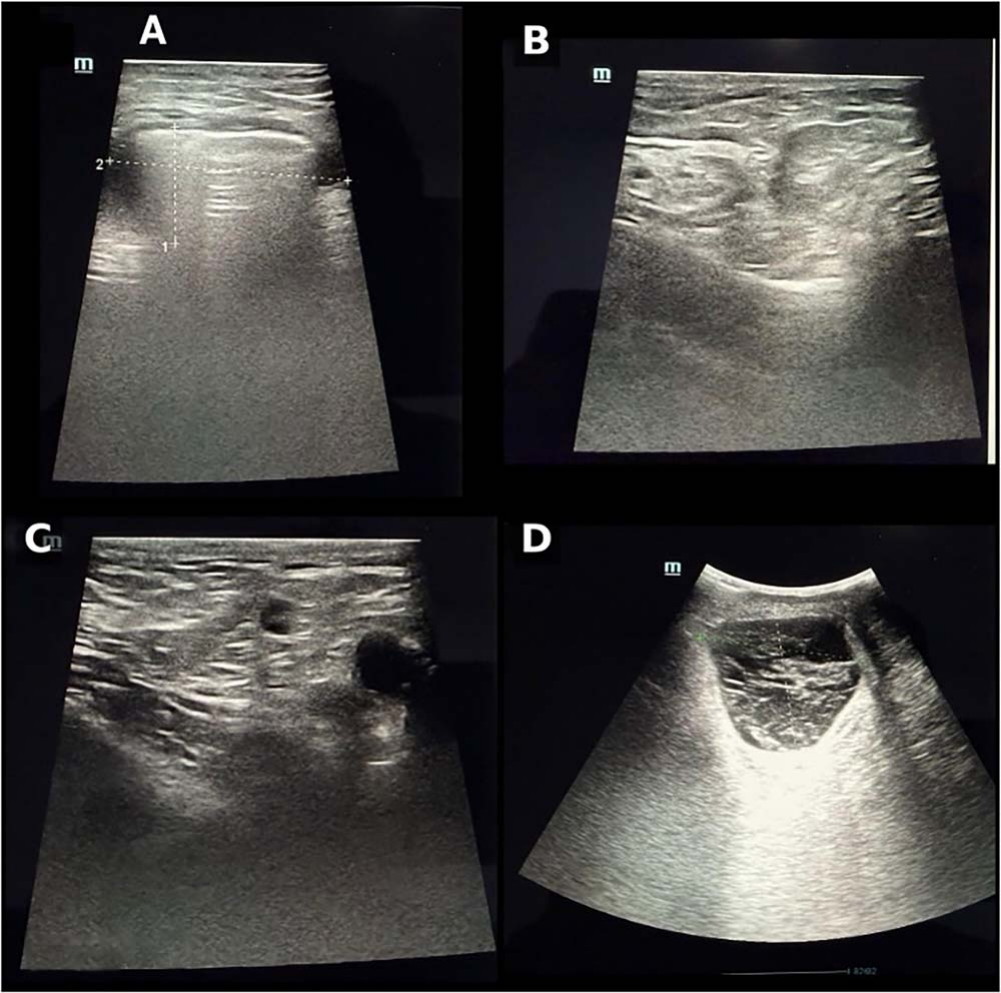
High-frequency linear-transducer soft-tissue ultrasound of the right inguinal region; (A) measurement of the herniary defect in the anterior abdominal wall, lateral to the epigastric vessels (55 × 63 mm); (B) heterogeneous fatty content and bowel loops within the sac, extending to the distal third of the inguinal canal; (C) herniary contents with hypoechoic foci compatible with intestinal gas; (D) convex-transducer view of a dilated bowel loop with mixed content inside the sac; the defect was neither reducible with directed transducer pressure nor modifiable with the Valsalva manoeuvre, without surrounding inflammatory changes.

He was haemodynamically stable on admission. The abdomen was distended and tender, with a non-reducible right inguinal defect and overlying skin discoloration. Laboratory tests showed neutrophilic leucocytosis, elevated C-reactive protein, and arterial blood gases with respiratory alkalosis and hyperlactataemia. Plain abdominal radiography revealed distended small-bowel loops with stepped air–fluid levels (Fig. 1). High-frequency linear ultrasound of the right inguinal region documented a 55 × 63 mm defect lateral to the epigastric vessels, containing fat and intestinal gas, extending to the distal third of the inguinal canal, neither reducible nor modifiable with the Valsalva manoeuvre (Fig. 2). The defect was classified as Nyhus IVB/European Hernia Society (EHS) L3R (lateral inguinal hernia, size 3 [>3 cm], recurrent).

**Figure 3:**
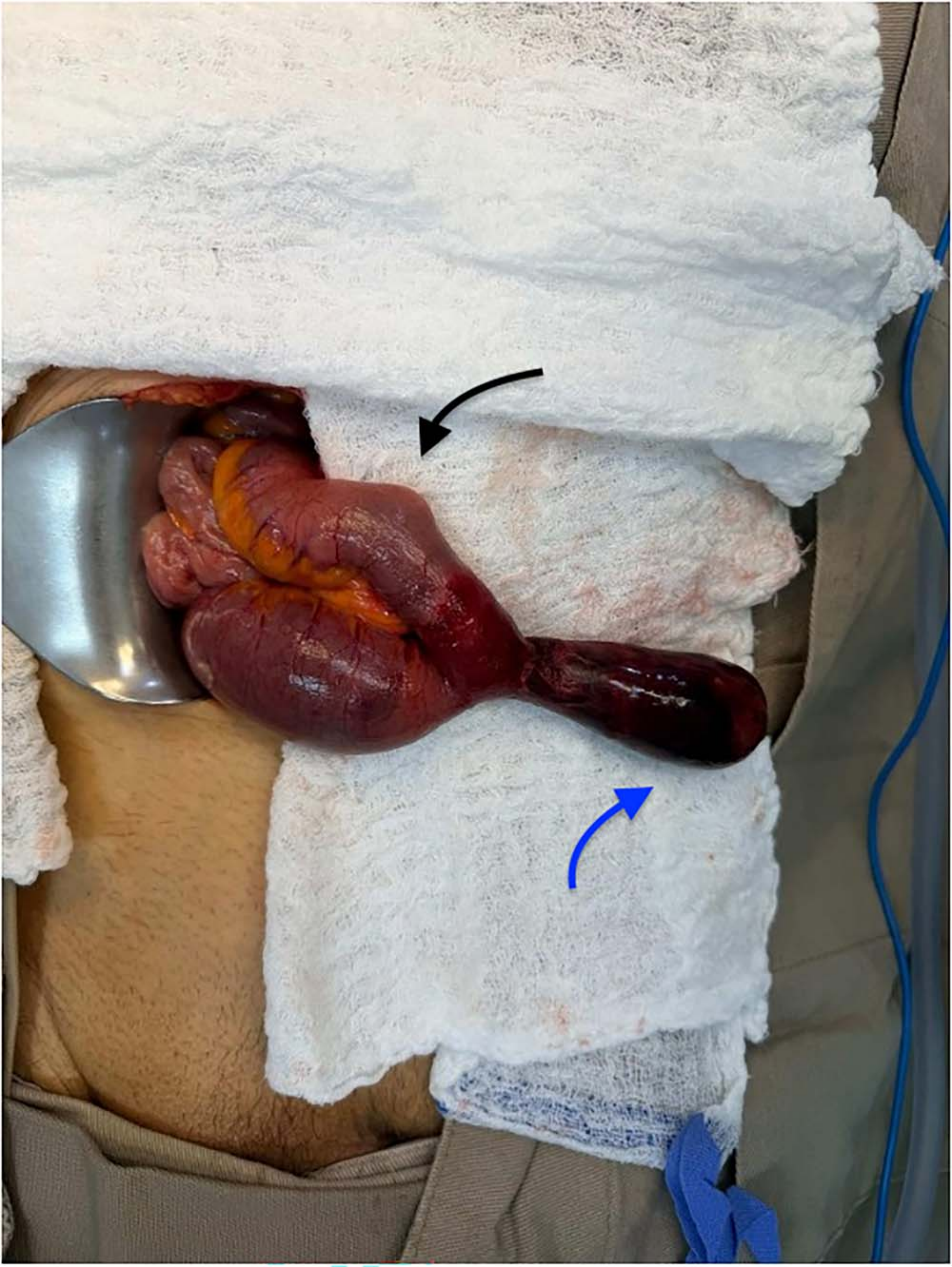
Intraoperative finding during infraumbilical exploratory laparotomy; arrow pointing to the Meckel’s diverticulum: approximately 10-cm Meckel’s diverticulum located 80 cm from the ileocaecal valve, strangulated within the recurrent right inguinal hernia sac, with diffuse venous congestion and ischaemic discoloration; arrow pointing to the ileal loop: adjacent distal ileal loop with ischaemic changes.

After fluid resuscitation, analgesia, broad-spectrum antibiotics, and nasogastric decompression with faeculent output, the patient underwent emergent infraumbilical exploratory laparotomy. A 10-cm Meckel’s diverticulum located 80 cm from the ileocaecal valve was found strangulated within a recurrent right inguinal hernia sac, together with an adjacent loop of small bowel—a mixed Littré’s hernia (Fig. 3). Segmental small-bowel resection with side-to-side linear stapled anastomosis was performed, followed by primary tissue repair of the defect without prosthetic reinforcement, given the contaminated field.

Histopathology showed a 6.0 × 2.0 cm ileal segment with an antimesenteric digitiform projection of 6.5 × 3 cm (10 cm *in vivo* before formalin fixation), of uniform 0.3-cm wall thickness, consistent with a true diverticulum. Non-perforated transmural ischaemic necrosis with acute serositis and viable resection margins was documented; no heterotopic gastric or pancreatic mucosa and no malignancy were identified.

Postoperative recovery was uneventful: transit resumed on Day 3, and the patient was discharged on Day 4 tolerating an oral diet, with no infectious complications or readmissions during early follow-up.

## Discussion

Littré’s hernia is exceptional, with fewer than 100 patients described worldwide [7]; each new case therefore adds practical surgical value. Meckel’s diverticulum, the most common remnant of the omphalomesenteric duct, is a true antimesenteric ileal diverticulum located 60–100 cm from the ileocaecal valve, with a prevalence of 0.3%–3% and a mean length of 5.4 cm [4, 7, 10]. The 10-cm diverticulum at 80 cm from the ileocaecal valve in our case exceeds the published average; larger diverticula are biomechanically more prone to protrude and incarcerate.

As a true diverticulum it contains all three intestinal layers and may harbour heterotopic mucosa—predominantly gastric—in about 15% of cases [7]. Its complete wall confers rigidity, providing an anchoring point that drags and compresses the adjacent loop within the hernia defect, as observed here. The Park criteria (age <50, male sex, length >2 cm, heterotopic tissue) predict up to a 70% complication rate when all four are present [11]; our patient met only two yet developed severe strangulation, illustrating their limited value when the index event is an acute mechanical complication. Mixed Littré’s hernia accounts for up to 34% of obstructive presentations [2].

Preoperative diagnosis is achieved in <10% of cases [8, 9]; abdominal-wall ultrasound is useful in selected scenarios [12] and computed tomography is preferred when a complication is suspected, although identification of the diverticulum within the sac is limited [9]. Mortality reaches 11.2% overall and up to 28% when obstruction is present [7], underscoring the need for prompt management—particularly in patients beyond the typical 56–60-year age range [6, 7]. Recurrent hernias add anatomical distortion and visceral compromise, which may explain the rapid progression observed despite a single day of symptoms [3, 13].

Two surgical decisions are required: management of the diverticulum and the involved bowel, and repair of the defect. Resection of the diverticulum within a hernia sac is universally recommended [1, 14]; when the adjacent bowel is compromised, segmental resection with primary anastomosis (stapled or hand-sewn) is rapid, safe, and well supported [4, 10]. Hernia repair in a contaminated field remains contentious: synthetic mesh is avoided because of infection and enterocutaneous-fistula risk [4, 10], so primary tissue repair was chosen. A comparable Colombian report described an 80-year-old woman managed by diverticulectomy and mesh repair in a clean-contaminated field [13]; our case differs by the coexistence of recurrence, strangulation, and frank ischaemia, which drove a more conservative strategy. Puentes *et al*. likewise highlight Meckel’s diverticulum in the differential of complex geriatric abdominal disease [15].

The principal limitation is its single-case nature. Nevertheless, this case supports a high index of suspicion in any complicated inguinal hernia in the elderly, systematic inspection of the sac contents, and the safety of primary tissue repair when strangulation, ischaemia, and contamination coexist. Littré’s hernia is uncommon, usually diagnosed intraoperatively, and lacks a standardised approach; timely recognition, rapid exploration, and individualised technique are decisive for the clinical outcome.

## Data Availability

All data underlying this case report are included within the manuscript. Additional anonymised data are available from the corresponding author upon reasonable request.
